# Prevalence and potential mechanisms leading to persistent elevation of high sensitive troponin T in patients 6 month after acute myocardial infarction

**DOI:** 10.1186/1532-429X-11-S1-P49

**Published:** 2009-01-28

**Authors:** Mirja Neizel, Tim Schaeufele, Grigorios Korosoglou, Dirk Lossnitzer, Harald P Kühl, Malte Kelm, Hugo A Katus, Evangelos Giannitsis, Henning Steen

**Affiliations:** 1 University Hospital Aachen, Aachen, Germany; 2 University Hospital Heidelberg, Heidelberg, Germany; 3 Department of Cardiology, Heidelberg, Germany

**Keywords:** Acute Myocardial Infarction, Acute Myocardial Infarction, York Heart Association Class, cTnT Level, High Sensitive Troponin

## Introduction

Persistent minor troponin elevation in patients with coronary heart disease is associated with adverse outcome and prognosis. However, the mechanism is not yet clearly understood.

## Purpose

Our objectives were to examine the prevalence and range of cardiac troponin T (cTnT) in stable patients six months after acute myocardial infarction (AMI) using a new high sensitive cTnT assay and to investigate the association of persistent minor cTnT-elevation to clinical variables, NT-pro BNP and cardiac MR-findings.

## Methods

cTnT was measured in 98 patients at 6 months after AMI. Patients were investigated in a clinical 1.5 Tesla whole body MR-scanner (Achieva, Philips, Best, the Netherlands) 3 ± 1 days after successful mechanical reperfusion of the infarct related vessel and at a follow-up of 6 ± 1 month using a 5-element phased array cardiac synergy coil.

Assessment of resting left ventricular function was determined by cine images using a steady-state-free precession sequence in 10–12 8 mm-thick slices covering the whole left ventricle from base to apex as well as long axis 2,-3 and 4-chamber views.

Ten minutes after gadolinium contrast injection (0.2 mmol/kg body weight of Gadolinium-DTPA (Magnevist^®^, Bayer, Germany)) late enhancement-imaging was performed using an inversion-recovery gradient-echo technique triggered to end-diastole. Data analysis of MRI-images was performed using a usual Philips work station.

cTnT was measured using a precommercial assay by electrochemiluminescence methods (Roche Diagnostics, Mannheim, Germany.

## Results

Minor cTnT-concentrations were detectable in 90% of the entire cohort, of whom 16% had cTnT-values above the 99^th^ percentile (> 12 ng/L). These patients were also significantly older, suffered more frequently from hypertension, had a higher New York Heart Association class and received more often diuretics at follow-up. Patients with cTnT-elevation had a more impaired left ventricular ejection fraction (p = 0.02) but did not have an increased infarct size (p = 0.73).

## Conclusion

Elevated minor cTnT levels are frequently detectable in patients 6 month after AMI. Increased cTnT-level were associated with clinical parameter for heart failure, impaired ejection fraction and higher NT-pro-BNP levels suggesting that myocardial dysfunction leads to persistent cTnT-elevation.

